# The Fracture Resistance of Endodontically Treated Teeth Restored Using 3 Different Posts: Protocol for a Comparative Evaluation Study

**DOI:** 10.2196/76630

**Published:** 2026-08-07

**Authors:** Sanika Damle, Manoj Chandak, Vinus Shivlani

**Affiliations:** 1 Department of Conservative Dentistry and Endodontics, Sharad Pawar Dental College and Hospital, Datta Meghe Institute of Higher Education and Research (Deemed to be University), Sawangi (Meghe), Wardha, Maharashtra, 442107, India, 91 07447401822

**Keywords:** fracture resistance, glass fiber posts, fiber-based post systems, endodontically treated teeth, comparative evaluation

## Abstract

**Background:**

Endodontically treated teeth are prone to fractures due to the loss of tooth structure, caries removal, access cavity preparation, and scaling, resulting in compromised strength in the teeth. Posts are used for restoring teeth that have undergone this process, but the role of different fiber posts in resisting fractures in teeth is unclear. Customized carbon fiber posts, customized glass fiber posts, and fiber posts relined with short fiber-reinforced composite (SFRC) are biomechanically efficient. SFRC is used as a relining material around the fiber post to improve adaptation to the root canal.

**Objective:**

This study aims to evaluate and compare the fracture resistance of endodontically treated teeth restored with prefabricated carbon fiber posts, customized glass fiber posts, and SFRC-relined fiber posts.

**Methods:**

In the current in vitro experimental study, the post specimens will be tested on 30 extracted single-rooted human teeth. These will be similar in size. The specimens will go through endodontic treatment and preparation for the post space, after which they will be randomly assigned to groups based on the type of post system: prefabricated carbon fiber posts, customized glass fiber posts, or SFRC-relined fiber posts. These will be cemented using a dual-cured resin cement. The specimens will then be restored using a standardized core build-up and tested for fracture resistance using a universal testing machine. A compressive load will be applied to each specimen at a constant crosshead speed of 1 mm/min until fracture of the specimen occurs. The maximum load at failure will be recorded in newtons. Statistical analysis will be conducted using a 1-way ANOVA.

**Results:**

Institutional ethics approval was granted by the Institutional Ethics Committee of Datta Meghe Institute of Higher Education and Research in January 2025 (DMIHER[DU]/IEC/2025/543). Sample collection and specimen preparation are scheduled to begin in February 2026, with mechanical testing expected to be completed by April 2026. As of April 2025, no specimens had been tested, and data analysis had not commenced. This is a nonfunded in vitro study, and data analysis is anticipated to be completed by May 2026. The results are expected to be submitted for publication by mid-2026.

**Conclusions:**

This study is expected to generate comparative biomechanical evidence on the fracture resistance of different fiber-based post systems, potentially aiding clinicians in selecting optimal restorative strategies for endodontically treated teeth.

## Introduction

### Background

Teeth that have been endodontically treated are particularly difficult to restore because they frequently lose a large portion due to decay, prior restorations, endodontic access cavity preparation, and the lack of moisture that dentin normally provides. Because of these alterations, the tooth becomes weaker and more prone to breaking. Post and core systems are frequently used to restore the strength and integrity of such teeth [1,2]. Over the past 10 years, cast posts and cores have been the most often used post type in dentistry. These typically need an extra laboratory step where a custom post is made based on the impression taken from the prepared post area [3-6].

Despite the long history and widespread use of cast metallic posts, certain issues have also been identified with these systems, such as corrosion, root fractures, loss of retention, the need to remove extensive root structure, and stress concentration [4]. Fiber post systems were introduced as a result of dental manufacturers searching for novel options due to these disadvantages. These systems’ reduced elastic modulus, which causes them to respond and exhibit stress patterns similar to those of dentin when subjected to external impacts, has been one of their most important characteristics [5].

For teeth that have had endodontic treatment, fiber-reinforced composite (FRC) posts have grown in popularity. Polymer matrix is reinforced with materials such as epoxy-based resins or other high-conversion polymers to create these posts. With a high degree of cross-linking adding to their endurance, the continuous fibers provide exceptional strength and resilience [6]. The milestone that altered some of the underlying concepts for restoration of teeth that had undergone endodontic procedures was the establishment of the first fiber posts in the dental field, known as carbon fiber-reinforced resin posts [7].

According to Duret et al [8], carbon fiber posts were initially composed of a matrix made up of 64% epoxy resin by weight. The first widely accepted substitute for cast posts, prefabricated metal posts, or zirconia posts was carbon fiber. Nevertheless, because carbon fiber posts are dark, they lack aesthetic appeal. At least in short-term trials, post-retained crowns made using carbon fiber posts have demonstrated qualities on par with and, in certain situations, superior to those of other prefabricated posts now in use [9]. Carbon posts’ strong flexural resistance means that less dentin structure needs to be removed, which helps prevent fractures of the post and the remaining tooth tissue [10].

A large volume by percentage of continuous unidirectional reinforcing fibers inserted in a polymer matrix is used to make prefabricated FRC posts. Usually composed of materials such as carbon, glass, or quartz fibers, these posts require little preparation before being placed [11]. Conversely, non-preimpregnated fibers, usually made of glass or polyethylene, are shaped into posts during the restoration process to make chair-side bespoke FRC posts [12]. For the treatment of severely damaged teeth brought on by cavities or trauma, custom-made posts and cores are sometimes advised. Because they fit precisely in the prepared post space, they are especially helpful for teeth with curved or elliptical canals [13].

Prefabricated posts, on the other hand, are inappropriate in these circumstances as they are unable to sufficiently conform to the canal’s curvature. Custom-cast post and core systems are more resilient to torsional stress because of their increased flexibility. Furthermore, these specific posts and cores act as stabilizers for the tooth’s crown and root sections in the case of single-rooted and premolar teeth, which have a tendency to deteriorate as a result of the gradual loss of tooth structure during preparation [14].

Short fibers (usually millimeters to a few centimeters long) are incorporated into a matrix material, usually a polymer, metal, or ceramic, to create a short FRC (SFRC).

When compared to long fiber composites, short fibers are more affordable and simpler to produce, and their goal is to improve the mechanical characteristics of the matrix, including strength, stiffness, and impact resistance [15]. Although carbon fiber posts have aesthetic drawbacks, SFRC and carbon fiber posts together can improve this aspect. The SFRC relining is a dependable and efficient option for root canal therapy as it also increases the restoration’s overall lifetime, offers long-term durability, and reduces the need for extensive tooth extraction during preparation [16].

As comparing the fracture resistance of different post systems is important for a number of reasons, particularly in dental restorations, this study will compare the fracture resistance of endodontically treated teeth restored using 3 different post types: prefabricated carbon fiber post, customized glass fiber post, and SFRC-relined fiber post [14,16].

Existing literature shows inconsistent and inconclusive evidence regarding the fracture resistance of different post systems due to variations in materials and study designs. This study aims to address this gap by providing a standardized, direct comparison of prefabricated carbon fiber posts, customized glass fiber posts, and SFRC-relined fiber posts.

### Objectives

The objectives of the present study are to evaluate the fracture resistance of endodontically treated premolar teeth restored using prefabricated carbon fiber–reinforced posts, custom-made glass fiber–reinforced posts, and SFRC-relined fiber posts and compare the fracture resistance of premolar teeth reinforced using these 3 post systems to determine their relative effectiveness in strengthening endodontically treated teeth.

## Methods

### Material Required

Thirty extracted human teeth will be chosen for this investigation. Teeth that have been extracted for orthodontic purposes and are free of cavities, cracks, or prior endodontic procedures will be used. Following extraction, the teeth will be cleaned of adherent soft tissue and calculus using an ultrasonic scaler and stored in 0.1% thymol solution at room temperature until further use to prevent dehydration and preserve dentin properties. Prior to experimentation, the teeth will be rinsed thoroughly using distilled water. To standardize length at 15 (± 1) mm, the teeth will be decoronated at the cementoenamel junction using a diamond disc mounted on a slow-speed handpiece under continuous water irrigation to minimize heat generation and prevent thermal damage to the dentin. A high-speed handpiece equipped with a round carbide bur (size 2) will be used to prepare straight-line access to the root canal. After that, a crown-down procedure will be used to instrument the root canals.

To clean the canal and remove debris, 3% sodium hypochlorite will be used as an initial irrigation solution for the root canals. The first cleaning will be conducted using a size 15 K-file (Dentsply Sirona). Using step-back instrumentation, the canal will be progressively extended up to a size 40 file. To remove germs and debris, extensive irrigation will be applied between each file. A size 40/.04 rotary file (ProTaper Next; Dentsply Sirona) will be used for the last preparation when the canal reaches the necessary size to guarantee that it is correctly tapered and well shaped. After removing the smear layer via a final irrigation using a solution of 3% sodium hypochlorite and 17% ethylenediaminetetraacetic acid, distilled water will be used for rinsing. Dia-ProSeal (DiaDent Group International) sealer and gutta-percha will be used for the obturating canal.

To preserve the apical seal, 4 mm of gutta-percha will be retained at the apical end of each root canal. The remaining coronal portion of the canal (approximately 10 mm) will be prepared as the post space by removing gutta-percha using Peeso reamers (Mani, Inc; 28 mm). The post space will then be refined using the manufacturer's corresponding black, red, and yellow drills.

The specimens will then be split into 3 groups and will be ready for post insertion once the sealant has solidified.

Using Prime etching gel (Prime Dental Manufacturing), the post space and the corresponding fibers will be etched and cleaned, and then a bonding agent will be applied, left to air dry, and then cured.

Following the etching and bonding technique described above, post selection and luting samples will be prepared in accordance with the appropriate groups.

For cementation, according to the manufacturer’s guidelines, resin cement (Calibra Universal self-adhesive resin system; Dentsply Sirona) will be used to cement the posts’ surfaces. To ensure that the sealer is equally distributed around the post, the posts will be carefully placed into the canals that have been created. After removing any excess cement, the posts will be light-cured from various angles for 20 seconds each to guarantee full polymerization. Every prepared root will be set inside a 15 × 15 mm acrylic resin block.

After mounting the specimens on a universal testing apparatus, the buccal surface will be subjected to a compressive load at a 90° angle to the long axis and a crosshead speed of 1 mm per minute until it fractures.

All the procedures—including access cavity preparation, canal instrumentation, post space preparation, post cementation, and core build-up—will be performed by a single trained operator following standardized protocols to minimize operator-related variability. Fracture resistance testing will be conducted using a universal testing machine calibrated before testing according to the manufacturer’s specifications, with a standardized crosshead speed applied until fracture. These measures will ensure methodological uniformity, reliability, and reproducibility of the study results. The force will be recorded in newtons.

### Statistical Analysis Plan

To evaluate variations in fracture resistance among the 3 groups, data will be analyzed using descriptive and inferential statistical methods. Descriptive statistics, including mean and SD, will be used to summarize fracture resistance values for each group. Intergroup comparisons of mean fracture resistance will be performed using 1-way and 2-way ANOVA. When a statistically significant difference is observed, the Tukey post hoc test will be applied for pairwise comparisons. Failure modes, being categorical variables, will be analyzed using the chi-square test. The Bonferroni adjustment test will be used for comparing different groups. Statistical analysis will be performed using the SPSS software (version 27.0; IBM Corp) and Prism (version 7.0; GraphPad Software), with the level of significance set at a *P* value below .05.

### Ethical Considerations

Ethics approval for this study has been obtained from the institutional review board of Datta Meghe Institute of Higher Education and Research (DMIHER) in January 2025 (DMIHER[DU]/IEC/2025/543). Extracted human teeth will be collected from the Department of Oral Surgery at DMIHER following orthodontic or nonpathological extractions, with prior written informed consent permitting their use for research purposes and ensuring no risk of harm to patients. All specimens will be handled in accordance with strict institutional and international ethical guidelines for the use of human tissues, with complete maintenance of confidentiality and compliance with the Declaration of Helsinki.

As this investigation will be conducted entirely as an in vitro study, registration with the Clinical Trials Registry - India will not be applicable and is therefore not required.

### Inclusion and Exclusion Criteria

The inclusion and exclusion criteria are outlined in Textbox 1.

Textbox 1. **Inclusion and exclusion criteria.**

**Inclusion criteria**

- Healthy human premolar teeth extracted for orthodontic or nonpathological reasons
- Teeth free from caries and prior endodontic treatment, with intact crowns and roots
- Single, straight root canal with standardized root length of 15 (± 1) mm
- At least 2 mm of coronal structure above the cementoenamel junction for post placement

**Exclusion criteria**

- Teeth with caries, cracks, fractures, or structural defects
- Premolars with multiple canals or previous endodontic treatment
- Teeth with severe root resorption or root length of <12 mm
- Teeth with inadequate coronal structure for post placement

### Sample Size

The sample size was calculated based on detecting a statistically significant difference in mean fracture resistance between 2 independent groups using previously published data.

The formula used for sample size estimation was as follows [17]:


$$n_{1}=\frac{(\sigma_{1}^{2}+\kappa \sigma_{2}^{2})(Z_{1-\alpha /2}+Z_{1-\beta }{)}^{2}}{\Delta^{2}}$$

In this equation, *n*_1_ represents the sample size per group, σ_1_ and σ_2_ represent the SDs of groups 1 and 2, Δ represents the expected difference in group means, κ represents the ratio of sample sizes between groups (assumed as 1), *Z*_1 – α/2_=1.96 represents the 95% CI, and *Z*_1 – β_=0.84 represents 80% power.

On the basis of the reference study by Thakur and Ramarao [1], the mean failure load was 179.75 (SD 33.52) N for group 1 and 146.44 (SD 13.53) N for group 2. The expected mean difference (Δ) was therefore 33.31 N.

Substituting the values into the formula yielded a minimum sample size of approximately 10 specimens per group. As the present study includes 3 experimental groups, a total sample size of 30 teeth (10 per group) was selected.

The power of the study was set at 80%, with a 5% level of significance (95% CI).

### Sample Allocation

A total of 30 single-rooted root canal treated teeth will be randomly divided into 3 groups (n=10 per group). Each group will be restored using a different post system: prefabricated carbon fiber post, customized glass fiber post, and SFRC-relined carbon fiber post.

## Results

### Overview

This is a nonfunded in vitro study. Sample collection and specimen preparation are scheduled to begin in February 2026, with mechanical testing expected to be completed by April 2026. At the time of manuscript submission, no specimens had been tested, and data analysis had not commenced. Data analysis is anticipated to be completed by May 2026. The results are expected to be submitted for publication by mid-2026.

### Primary Outcome

The primary outcome of this study is expected to show that SFRC-relined fiber posts exhibit the highest fracture resistance due to their superior mechanical properties, including enhanced fracture toughness and flexural strength. Customized glass fiber posts are anticipated to perform better than prefabricated carbon fiber posts as their customized fit ensures optimal adaptation to root canal anatomy, leading to improved stress distribution and reduced adhesive failure. Prefabricated carbon fiber posts, while offering good fracture resistance, may perform slightly lower than the other 2 systems, particularly in nonideal root canal shapes.

## Discussion

Several studies have explored the fracture resistance of teeth that have undergone endodontic treatment and been restored using various post systems. Jakkamsetty et al [3] found that teeth reinforced with prefabricated hybrid posts exhibited the highest fracture resistance, significantly outperforming prefabricated glass fiber posts, prefabricated threaded metal posts, and teeth restored without posts. These findings suggest that hybrid posts provide superior structural support for extensively damaged endodontically treated teeth. These findings suggest that hybrid posts provide superior structural support for teeth with significant damage, highlighting their effectiveness in postendodontic restorations. Thakur and Ramarao [1] conducted a study evaluating different post systems and found that prefabricated glass fiber posts showed comparatively higher fracture resistance than polyethylene-woven fiber posts, glass fiber posts, and prefabricated carbon posts. The post length, whether half or two-thirds the root length, did not significantly affect fracture resistance. This suggests that, while glass fiber posts provide superior fracture resistance, post length may not play a major role in enhancing the fracture strength of endodontically treated teeth. Such results emphasize the necessity of post material over length in ensuring restoration durability [1]. Adanir and Belli [2] conducted an in vitro study that demonstrated that posts shorter than the clinical crown length significantly reduced root fracture resistance. No significant difference was found between different cement types used for post cementation. The latter study concluded that posts shorter than clinical crown length should not be used as they result in lower fracture resistance, increasing the risk of clinical failure. Such results emphasize the importance of appropriate post length for achieving optimal root restoration strength [2].

Although previous studies have compared various fiber post systems, the literature shows inconsistent results regarding the most effective post design for reinforcing endodontically treated premolars [18,19]. Direct comparative evidence among prefabricated carbon fiber posts, customized glass fiber posts, and SFRC-relined fiber posts under identical experimental conditions is limited [20,21]. This study aims to address this gap by providing a standardized, head-to-head comparison of these 3 post systems to clarify their relative fracture resistance and clinical relevance.

As this is an in vitro study, it does not fully replicate real oral conditions such as occlusal forces, saliva, and temperature variations, which could influence the bond strength and fracture resistance of the tested posts. Additionally, material variability due to differences in composition and manufacturing processes may introduce inconsistencies, making it difficult to generalize findings across all commercially available products [15,22,23].

Factors such as thermal cycling, the presence of saliva, variations in intraoral humidity, and dynamic occlusal forces could not be simulated in the current experimental setup. Consequently, the fracture resistance values obtained may not fully replicate the complex biomechanical behavior of restored teeth under functional intraoral conditions [24-26]. Therefore, the results of this study should be interpreted with caution, and future investigations incorporating thermomechanical aging and simulated oral conditions are recommended to enhance the clinical relevance and translational value of the findings [27,28].

The present study aims to compare the fracture resistance of endodontically treated teeth reinforced with 3 different post systems: prefabricated carbon fiber post, customized glass fiber post, and prefabricated fiber post relined with SFRCs. The key focus is to evaluate how the material and configuration of posts impact fracture resistance under controlled loading conditions.

The study will assess (1) fracture resistance by measuring the mean failure load of each post system using a universal testing machine and (2) clinical relevance by providing insights on the best post material and design for reinforcing endodontically treated teeth, guiding clinicians in material selection for long-lasting restorations.

## Abbreviations

DMIHER: Datta Meghe Institute of Higher Education and Research
FRC: fiber-reinforced composite
SFRC: short fiber-reinforced composite
